# Tuberculosis infection and epidemiological characteristics in Haidian District, Beijing, 2005–2018

**DOI:** 10.1186/s12889-020-08773-8

**Published:** 2020-06-01

**Authors:** Fan Wu, Caiyun Lai, Yan Wang, Gaoqiang Zhang, Yueqi Li, Susu Yu, Xinyue Peng, Jiani Yang, Zhisheng Wei, Wenjuan Zhang

**Affiliations:** 1 Department of Public Health and Preventive Medicine, School of Medicine, Jinan University, Guangzhou, Guangdong 510632 People’s Republic of China; 2 Department of preventive health care, Four Seasons Hospital, Haidian District, Beijing, 100097 People’s Republic of China

**Keywords:** Tuberculosis, Epidemiology, Etiological diagnosis, Beijing

## Abstract

**Background:**

This study was aimed to investigate the epidemiological characteristic of pulmonary tuberculosis (PTB) in Haidian District, Beijing from 2005 to 2018 and to provide suggestions for controlling tuberculosis (TB) development.

**Methods:**

Epidemiological data about TB were obtained by the Infectious Disease Reporting System at different levels of medical institutions in Haidian District of Beijing from 2005 to 2018. The epidemiological methods combined with χ^2^ test were used to analyze the distribution of TB in population, time, region and TB diagnosis.

**Results:**

In total, 14,449 cases of TB patients were reported in Haidian District from 2005 to 2018 and the average annual morbidity was 31.67/10,000. Of the total cases, housework and unemployed people (20.73%; 2996/14,449) accounted for the highest proportion of occupational distribution, followed by students, accounting for 17.18% (2482/14,449). 2433 patients with the age of 65 years and over accounting for 16.83% (2433/14,449); Laboratory confirmed diagnosis of TB was 26.60% and the diagnostic delays accounted for 54.96%.

**Conclusions:**

From 2005 to 2018, TB incidence was falling gradually in Haidian District. However, particular attention should be paid to the elderly and student groups, and the policy publicity and education should be strengthened to reduce the diagnosis delay of TB.

## Background

Tuberculosis (TB), an ancient infectious disease caused by the bacillus *Mycobacterium tuberculosis*, have affected humans for thousands of years. It is the ninth leading cause of death worldwide and the leading cause of death by a single infectious agent, ranking above HIV/AIDS. Millions of people are infected with TB each year according to the World Health Organization (WHO) [1]. TB may spread from ill person to healthy person through the air via coughs, sneezes and spit.

It was estimated that 10 million people fell ill with TB in 2017 in the world. China was one of the 30 countries with a heavy burden of TB, accounting for 9% of TB patients, with 63 deaths in every 100,000 people. Mozambique, Philippines and South Africa are the three countries with the highest burden of TB, with more than 500 cases of every 100,000 people. Specific targets for 2030 set in the End TB Strategy would reduce 90% in the absolute number of TB deaths and 80% in TB incidence (new cases per 100,000 people per year), compared with those in 2015 separately [1].

In 1991, the Chinese government introduced Directly Observed Treatment, Short-course (DOTS) strategy, and created the national TB control system, with TB dispensaries and medical institutions at all levels, including rural primary health service networks and urban community health service institutions [2]. Especially, after the Severe Acute Respiratory Syndrome (SARS) was brought under control in 2003, the government increased public-health funding, revised laws of infectious diseases control, implemented the world’s largest internet-based disease reporting system, and started a programme to rebuild local public-health facilities in China [3].. The report of the fifth national sample survey pointed out that the prevalence of TB in eastern China in 2020 had declined compared with that in 2000 [4]. With timely diagnosis and correct drug treatment, most people with TB could be cured, but there are still many people falling ill TB every year, bring the great health and economy burdens.

The average resident population in Beijing is 21,640,000, Haidian District is located in the west and northwest of Beijing city, with an annual resident population of 3,260,000. It has the highest GDP among sixteen districts. There are many colleges and tourists attractions, such as the famous Peking University, Tsinghua University, the Summer Palace and the Zhongguancun Science Park in China. With the prosperous economic and educational environment, it attracted a large number of immigrants and floating people from different regions of China and other countries. About 344,400 foreigners visit the Haidian District every year. As different populations are prone to gather here, the disease spread easily. Therefore, we collected the TB data of Haidian District and analyzed the epidemiological characteristics to provide the theoretical basis and data reference for its prevention and control in Beijing, China.

## Methods

### Data inclusion

The diagnosis of TB is based on etiology (including bacteriology and molecular biology), combined with clinical manifestations, chest imaging, epidemiological reasons, and other auxiliary examinations for comprehensive analysis. TB cases were based on ≥1 of the following diagnostic criteria, including sputum or body fluid and tissue of smear-positive for acid-fast bacilli (AFB), culture-positive of *Mycobacterium tuberculosis* complex, or both; or clinical appearance, radiographic appearance, or both. A patient > 2 initial sputum smear examinations (direct smear microscopy) AFB-positive; or 1 sputum examination AFB-positive plus radiographic abnormalities, symptoms, or both consistent with active PTB was diagnosed as smear-positive PTB (SPPTB). The diagnosis of smear-negative PTB (SNPTB) case predominantly was relied on clinical symptoms (such as cough for > 2 weeks, fever for > 2 weeks, weight loss, hemoptysis) together with abnormal chest radiograph, the results of bacterial culture, and the response to diagnostic anti-TB treatment.

### Classification methods and definitions

TB has appeared among all ages including children under 14 years old, so we divide the age into eight stages with the interval of 14. There were 19 occupations in the information system report, among which cadres staffs refer to the employees working in public institutions; Housework are those without a formal job and the unemployed mean people who are unemployed and were reported as such by the respondents; Others refer to people holding occupations other than those listed. Workers were those patients reported to be employed for physical or technical work to earn wages. Migrant workers refer to those individuals who came from rural areas, but engaged in non-agricultural work in cities. Unknowns meant without the occupation report in detail. The delay in diagnosis was defined as more than 2 weeks from the reported onset of TB symptoms to diagnose at a hospital.

### Data source

The information of TB patients came from the Chinese Tuberculosis Management Information System. The regional distribution data of Haidian District were derived from the National Bureau of Statistics, and the population data came from Beijing statistical yearbook. Data used in this study were collected from records on condition of anonymity.

### Statistical analysis

Categorical variables were summarized as proportions including sex and patients type (SPPTB or SNPTB). The continuous variables (age) was summarized with mean and standard deviation (mean ± SD). The Chi-squared test was used to assess the difference in categorical variables. All analyses were performed using SPSS software (version 16.0). The criterion was *P* < 0.05, based on two-sided tests for statistical significance.

## Results

### The epidemic situation of TB cases

There were 14,449 TB cases during 2005–2018 in Haidian District, with an average annual incidence rate of 32.67/100,000. The new occurrences were 14,371 (99.46%; 14,371/14,449) and the treated/recurrent cases were 78 (0.54%; 78/14,449) as shown in Table 1. Meanwhile, there were 134 cases of combined pleurisy and 2 carriers of pathogens in 2005. The 5 rifampicin-resistant cases were reported in 2017 and 25 rifampicin-resistant cases in 2018 respectively. The morbidity of TB was the highest in 2009 with an incidence rate of up to 41.63/100,000 and this has begun to decrease since 2009 until a small increase in 2017. The lowest annual incidence was 26.01/100,000 in 2007.

**Table 1:** The morbidity and case type of TB patients in Haidian District, 2005–2018

Year	no. patients	Morbidity (/100,000)	Case type		TB pleurisy
			New cases	treated cases or recurrence	
2005	986	38.13	983	3	27
2006	767	28.54	754	13	19
2007	732	26.01	723	9	7
2008	912	31.13	891	21	9
2009	1283	41.63	1279	4	2
2010	1204	36.70	1200	4	6
2011	1251	36.77	1242	9	9
2012	1228	35.25	1224	4	3
2013	1118	31.26	1116	2	6
2014	1057	28.74	1055	2	6
2015	1036	28.05	1032	4	6
2016	965	26.86	965	0	5
2017	1011	29.05	1010	1	13
2018	899	26.77	897	2	16
Total	14,449	32.67*	14,371	78	134

*Table footnotes: The average annual morbidity

### Population distribution analysis

As summarized in Table 2, the number of TB was 1.69 times greater in males than in females from 2005 to 2018 in Haidian District. The number of males was 9083 with the annual average morbidity of 49.09/100,000, while the number for females was 5366 with the annual average morbidity of 31.42/100,000. There was a significant sex difference the incidence (*P* < 0.001). The average age of these patients was 39.69 years. The number of young people, aged from 15 to 24, was highest with a total of 4380 cases (30.31%; 4380/14,449), followed by those who were 25 to 34 years old with a total number of 3626 (25.10%; 3626/14,449). Only 0.41% (59/14,449) of total of TB patients were 14 years old group or less. The oldest patient was 101 year of age and the youngest one was only one day old. The significant difference was observed in each age group (*P* < 0.001). In terms of different occupations, the housework and unemployed individuals comprised 2996 cases, accounting for 20.73% (2996/14,449) and the students up to 2482 cases, occupying for 17.18% (2482/14,449).

**Table 2:** The patient demographics of TB in Haidian District, Beijing, 2005–2018

Characteristics	f	(%)	*p*-value
Age,(years)			
0–14	59	0.41	
15–24	4380	30.31	
25 ~ 34	3626	25.10	
35 ~ 44	1481	10.25	< 0.001
45 ~ 54	1306	9.04	
55 ~ 64	1164	8.06	
65 ~ 74	976	6.75	
≥ 75	1457	10.08	
Sex			
Female	5366	37.14	
Male	9083	62.86	< 0.001
Career			
Housework and unemployed	2996	20.73	
Students	2482	17.18	
Retired staffs	2189	15.15	
Cadre staffs	1960	13.56	
Others	1028	7.11	
Business servicers	921	6.37	
Workers	835	5.78	
Farmers	513	3.55	
Unknown	422	2.92	
Food and beverage industry workers	273	1.89	
Migrant workers	239	1.65	
Teachers	215	1.49	
Medical staffs	167	1.16	
Public place attendants	163	1.13	
Scattered children	18	0.12	
Seafarers and long distance drivers	18	0.12	
Nurses and babysitters	5	0.03	
Kindergarten children	3	0.02	
Herders	2	0.01	
Total	14,449	–	

### Local distribution

**Fig. 1:**
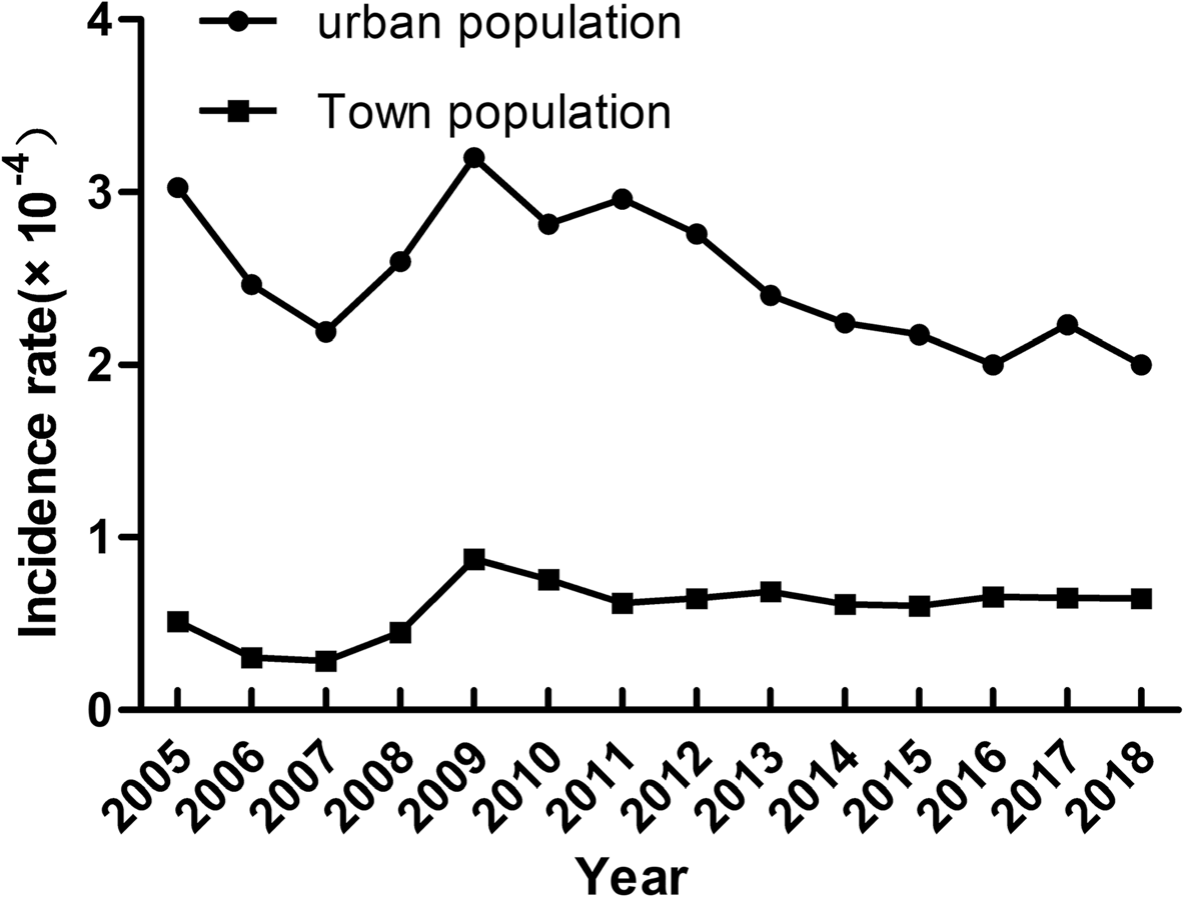
Regional distribution of TB. The incidence rate of TB in two different populations, including the local urban and town populations in Haidian District of Beijing, 2005–2018

TB cases occurred in 22 streets and 7 towns in Beijing Haidian District during the investigation period. The total number of cases was 11,359 in The 3843 cases were diagnosed solely as laboratory-confirmed and 2761 in towns, accounting for 78.6% (11,359/14,449) and 19.1% (2761/14,449) respectively. Additionally, there were 329 persons with unknown addresses, occupying 2.3% (329/14,449) of the total number of cases. The incidence rate was the highest in 2009 of urban population, and has been declining gradually since then. However the incidence rate of town population increased gradually from 2005 to 2009, and remained stable thereafter (Fig. 1).

### Etiological diagnosis

**Fig. 2:**
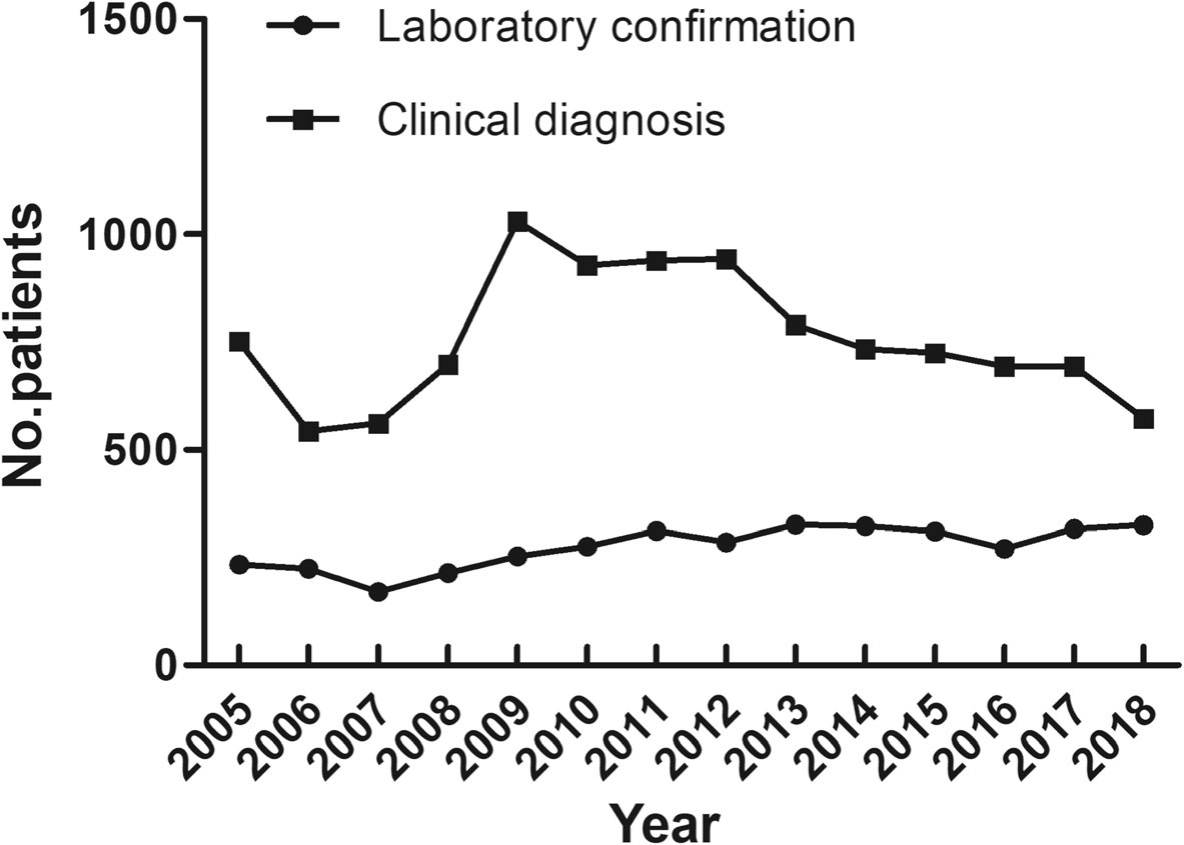
The number of the laboratory confirmation and clinical diagnosis reported TB patients in the Haidian District, Beijing, 2005–2018

The 3843 cases were diagnosed solely as laboratory-confirmed cases, and 10,606 cases were determined by radiographic appearance and laboratory diagnosis as the clinical cases, accounting for 26.6% (3843/14,449) and 73.4% (10,606/14,449) of the total cases respectively, of which 134 had pleurisy. The clinical diagnosis exhibited a downward trend, while the laboratory confirmation showed an upward trend from 2005 to 2018 from Fig. 2. The highest positive rate occurred in 2018 and the number of smear positives highest in 2011. There was a total of 3849 bacteria positive cases including smear-positive, positive bacterial culture and Rifampin resistance. The bacteria negative reached 7344 cases and the average rate of sputum was 77.47% (11,193/14,449) (Table 3).

**Table 3:** Diagnosis type for reported TB cases in Haidian District, 2005–2018

Year	Bacteria positive			Bacteria negative (cases)	Without sputum examination (cases)	Total	
	Swear-positive TB (cases)	Positive bacterial culture (cases)	Total (cases)			Positive rate (%)	Rate of sputum (%)
2005	179	6	185	440	361	18.76	63.39
2006	165	27	192	391	184	25.03	76.01
2007	169	1	170	379	183	23.22	75.00
2008	196	18	214	504	194	23.46	78.73
2009	272	10	282	716	285	21.98	77.79
2010	260	22	282	675	247	23.42	79.49
2011	314	29	343	612	296	27.42	76.34
2012	281	25	306	624	298	24.92	75.73
2013	299	34	333	548	237	29.79	78.80
2014	288	35	323	533	201	30.56	80.98
2015	283	27	310	501	225	29.92	78.28
2016	250	19	269	487	209	27.88	78.34
2017	280	37	317	514	180	31.36	82.20
2018	251	72	323	420	156	35.93	82.65
Total	3487	362	3849	7344	3256	26.64	77.47

### Diagnosis time

**Fig. 3:**
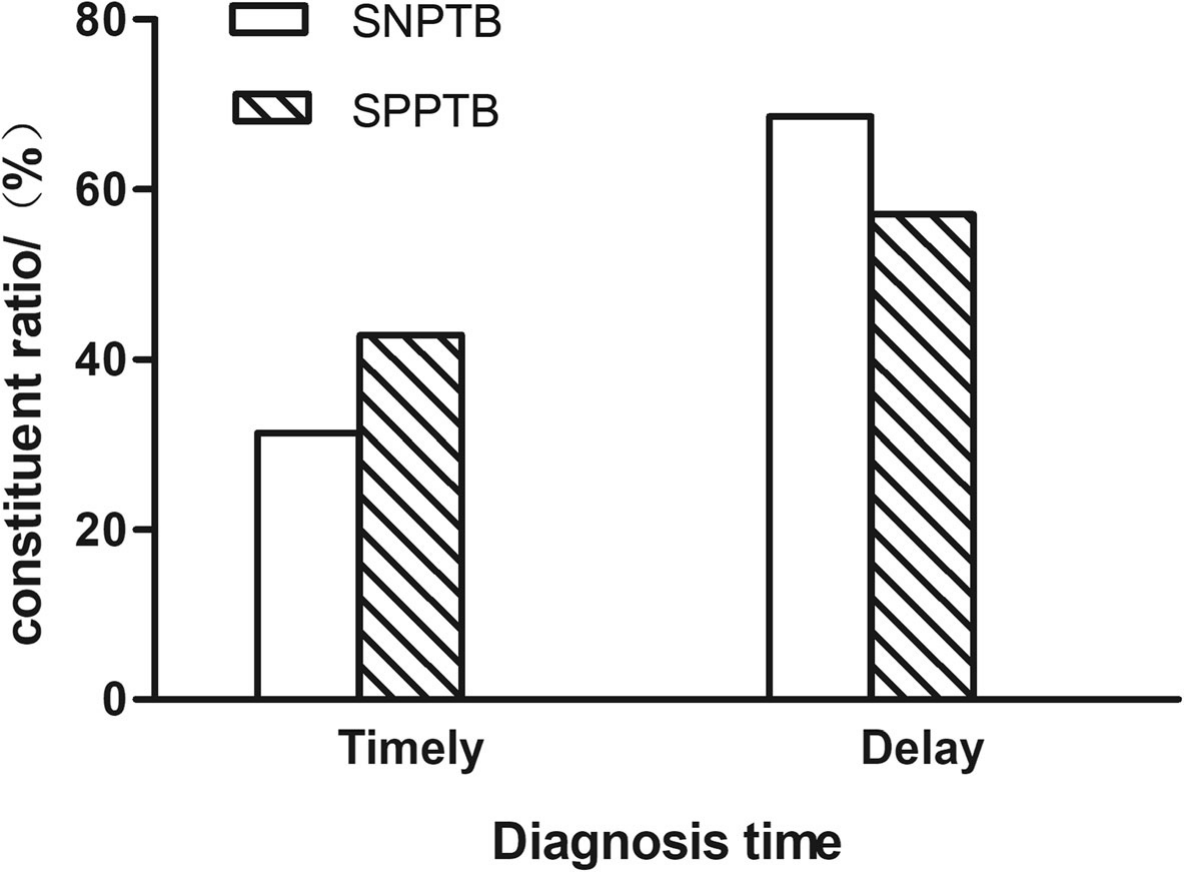
The diagnosis time (in weeks) of TB. Differences in the constituent ratio between timely diagnosis and delayed diagnosis in SNPTB and SPPTB of reported TB cases in Haidian District, Beijing, 2005–2018

As illustrated in Fig. 3, the average diagnosis time was 56.41 days with the median of 18 days. The average diagnosis time was 55.72 days for smear positive. Among them, 6 cases were diagnosed in advance and the earliest was 1 year. The 7941 people showed a diagnosis delay between 2005 and 2018, accounting for 54.96% of the total case. Among the delayed diagnosis cases, 1591 cases were smear-positive and 5115 cases were smear-negative, with the delay rates of 20.04% (1591/7941) and 64.41% (5115/7941) respectively. The smear-positive diagnosis delay rate was less than the smear-negative with the statistically significant difference (*P* < 0.001).

## Discussion

In this study, the TB morbidity was decreased by 29.8% (from 38.13 to 26.77 per 100,000 population) in Haidian District of Beijing from 2005 to 2018 with an average annual morbidity of 32.67/100,000, closely to the total incidence of TB reported in Beijing in 2017 (32.70 per 100,000 population) [5], but far lower than that in China in 2017(63 per 100,000 population) [1], which was closely related with the national policies to control the incidence of TB. Since 1949, China has paid much attention to the prevention and control of TB. The Chinese government incorporated this issue into the economic and social development plan, continuously increased the investment of prevention and control funds, strengthened the construction of institutions, and constantly improved the prevention and control service system. There was an important period of improvement and innovation in TB control from 2001 to 2010. By the end of 2005, China’s DOTS coverage had reached 100% [6].

From the perspective of age distribution, most patients were young adults aged from 15 to 44. However, the increasing attention should be placed on the old population over 75 years old. Many relapsed patients were found in the elderly population [7]. The immunity of the elderly population has generally weakened, and hence prone to infection or relapse. Additionally, some investigations showed that the BCG vaccine was more useful for younger people and less effective for the middle or senior-age people, with an average efficacy of only about 50% for those groups [8]. Considering the rapid aging of the Chinese population and high morbidity rate of TB in seniors, we should pay more attention to the potential high-risk elderly sub-populations who may contribute to the increased proportion of the actively infected people [9]. In this context, an effective vaccine control strategy should be implemented for them to reduce their TB morbidity.

The results showed that the most cases were distributed in urban areas, where a number of colleges and universities were located, As a consequence, students were found to be a high-risk group of TB. TB was easy to spread among crowed population, leading to the prevalence in campus areas. Schools are considered common places for the community-based of outbreak of TB in China, so the exposure risk in the dorm room and tuberculin test results should be taken into consideration in prevention and control [10, 11]. Moreover, although students were screened, no surveillance, follow-up or control activities were carried out, which may result in delays in the diagnosis of TB and thus caused its wide spread [12]. Therefore, the delayed diagnosis, lacked of preventive treatment and no follow-ups were the typical contributing factors in the outbreak of school TB.

Our study found that the incidence of TB in males was higher than that in females, and the occupational distribution of TB was mainly due to housework and unemployment and students. These were well explained by cluster aggregation. Men may have a higher morbidity of latent TB infection and greater exposure to conditions that favor the development of the disease, such as alcoholism, smoking and precarious working conditions [13, 14]. In the decade after the outbreak of SARS in China, TB incidence showed an increasing trend by age, especially noticeable among men [15].

From 2005 to 2018 in Haidian District, the positive rate of laboratory detection was lower than the 50% positive rate of pathogen diagnosis proposed in the 13th Five-Year National Tuberculosis Control Plan. Meanwhile, the smear-positive diagnosis delay rate was significantly less than that of the smear-negative diagnosis. Therefore, the laboratory testing level should be strengthened, and conventional smear culture should be combined with genetic testing to improve the detection rate of pathogens in Haidian District in the future. In particular, medical staffs should improve the vigilance of smear-negative patients with suspicious symptoms to avoid the diagnosis delay.

Although DOTS have been fully covered, there are still delays in TB diagnosis which may be caused due to the long distance from home to the health center and the physical problem of many elderly people who have difficulty in walking. Housework and the unemployed, many of whom lived in rural areas and were older, also had this problem. Additionally, with the amount of housework and farm work, TB patients in rural villages felt it almost impossible to visit the long distant health centers. Even though TB diagnosis and treatment are free, they may not be able to afford the costs associated with other things in the diagnosis process such as accommodation, dinning. Although most of the patients knew TB disease, they were lacking in knowledge about the symptoms and few people were aware of its severity. Discrimination from others may also discourage them to have access to the healthcare services. All these factors contribute to the delays in diagnosis, so resulting in the high prevalence of TB among the elderly, housework and unemployed [16].

In addition to causing the parenchyma of the lung, mycobacterium TB affects other areas other than the lung, leading to extrapulmonary tuberculosis (EPTB), most commonly pleurisy. EPTB is usually not an infectious disease. However, it can cause death if undiagnosed or untreated, especially in immunosuppressed individuals. EPTB patients were largely at 65 years or older, who had retired and was living in urban areas [17]. In our study, TB pleurisy has been reported every year. Notably, patients with HIV and diabetes were more likely to contract TB, but less likely to contract EPTB [18].

### Strengths and limitations

In this study, we have carried out a detailed descriptive epidemiological study to provide data references for the TB control. However, there are still some limitations to our study. In the future, the qualitative methods should be applied to explore the reasons for delay in diagnosis, including studies on individual behaviour and characteristics embedded in social, cultural and health service. In addition, a large scale study to cover the whole of Beijing might be beneficial.

## Conclusions

In conclusion, the overall incidence of TB in Beijing Haidian District dropped from 2005 to 2018, but there were still many new cases every year. National plans for TB control need to target populated regions, with special attention to elderly males without occupation who are at higher risk, and should strengthen publicity and education activities, especially in schools.

## Data Availability

Data of the study was not publicly available, the datasets used and analyzed during the current study are available from the corresponding author upon reasonable request. Restrictions apply to the availability of these data, which were used under license for the current study, and so are not publicly available. Data are however available from the authors upon reasonable request and with the permission of Four Seasons Hospital.

## Abbreviations

TB: Tuberculosis
DOTS: Directly Observed Treatment, Short-course
AFB: Acid-fast Bacilli
PTB: Pulmonary Tuberculosis
SPPTB: Smear-positive Pulmonary Tuberculosis
SNPTB: Smear-negetive Pulmonary Tuberculosis
EPTB: Extra-pulmonary Tuberculosis
SARS: Severe Acute Respiratory Syndrome
